# Microbes on the edge of Occam’s razor

**DOI:** 10.1186/s13062-014-0025-6

**Published:** 2014-11-30

**Authors:** Petro Starokadomskyy

**Affiliations:** Department of Internal Medicine, UT Southwestern Medical Center, 5323 Harry Hines Blvd, Dallas, Texas 75235 USA

**Keywords:** Microbes, Religious rituals, Microbiome-brain axis, Biomeme hypothesis, Popper, Falsifiability, Occam’s razor

## Abstract

**Abstract:**

Our body harbors hundreds of microbial species and contains many more bacterial than human cells. These microbes are not passive riders but rather a vital component of the organism. The human microbiota affects our health in multiple ways, both positively and negatively. One of the new attractive directions in microbiome biology is the “microbiome-brain axis”. Several groups of researchers have described the ability of the gut microbiota to communicate with the brain and thus modulate human behavior. These limited experimental data became the foundation of the “biomeme hypothesis” of possible microbial origin of some religious rituals that has recently appeared in Biology Direct. Here I propose a critical analysis of this hypothesis. I conclude that there is no evidence of the microbial origin of religious practices but there are strong indications of their psychological and social roots.

**Reviewers:**

This article was reviewed by Eugene Koonin, Neil R Smalheiser, Etienne Joly.

## Background

There seem to be no “sacred cows” for scientific inquiry anymore as essentially any entity, phenomenon or process can be dissected and quantitatively investigated with modern technologies. Today, using large quantitative data sets from hundreds of genomic and proteomic screens available in open access databases, scientists can generate a variety of novel hypotheses and full-fledged theories without even running “wet” experiments [1-5]. In line with this scientific paradigm, Panchin et al. [6] posed an interesting question: what if some microbes might subtly manipulate human brain toward certain religious rituals that are beneficial for the propagation of the given microbe(s). They called it the “biomeme hypothesis”. Under this hypothesis microbes do not induce religiosity as such but rather incline individuals toward particular religious rituals. In the concluding remarks the authors suggest that additional types of behavior also might be influenced by microbes but some religious rituals are the best markers of such effects because participation in them provides no apparent benefits to anyone but the hypothetical microbes.

The idea of cross-interaction between the host and its microbiota certainly is worth attention. Indeed, the existence of a “microbiome-brain axis” is well defined in several works [7,8], and such data can provide some indirect and speculative evidences for a possible role of microbes in a human religious behavior. For instance, germ-free mice exhibit less anxiety and fear [9]: two factors that potentially might be important driving forces of human religiosity. Another example is provided by *Toxoplasma gondii* that is suspected to be guilty in shifting of a number of behavioral characteristics of multiple hosts [10]. Hence, theoretically microbes have a potential to modulate people’s behavior such as to make them be more inclined to take part in religious rituals. However, there are hundreds of other reasons for people seeking collective spiritual experiences, and these reasons are traditionally studied by psychology and sociology rather than microbiology.

Do we really need to introduce a microbial component for understanding why people participate in religious rituals? In order to answer this question, I analyzed Panchin’s hypothesis using two well-known logical approaches. Firstly, I analyzed whether the biomeme hypothesis complies with the definition of a scientific hypothesis. Secondly, I evaluated a potential redundancy of the biomeme hypothesis using Occam’s razor. In conclusion, it appears that the individual decision to participate in religious rituals is driven by factors that are rooted in human psychology not in the microbiome.

## Unfalsifiability of the biomeme hypothesis

The philosopher Karl Popper has introduced the term “falsifiability” as a key parameter for demarcation of a scientific hypothesis from a non-scientific one [11]. Falsifiability is the logical possibility that a hypothesis can be proven false by a particular observation or experiment. By Popper’s criterion, unfalsifiable statements are non-scientific. Although non-scientific hypotheses may still have relevance, these cannot claim to be backed by scientific data, and therefore should be considered speculative and too weak to opposite the existing genuine scientific hypotheses.

The hypothesis of Panchin et al. certainly does not pass Popper’s test. Originally they propose (highlights in bold by PS): “*We hypothesize that ***certain aspects of religious behavior ***observed in the human society could be influenced by microbial host control and that the transmission of ***some religious rituals*** could be regarded as the simultaneous transmission of both ideas (memes) and parasitic organism.”* [6]. Theoretically, this statement can be falsified under either of two conditions:

1. The known microbes incline people to participate in some undefined religious rituals;
2. Unknown microbes incline people to participate in given religious rituals.

However, the biomeme hypothesis becomes irrefutable when neither particular rituals nor microbes are strictly defined. Panchin’s hypothesis as such postulates a nonsense: unknown microbes influence unknown aspects of religious behavior. This is similar to trying to solve a single linear equation with two variables.

Lack of knowledge of the critical parameters makes the biomeme hypothesis untestable. For instance, to define culprit microbes the authors have proposed to perform a next-generation microbiome sequencing of samples obtained from *gut or brain of people with a history of voluntary active participation in certain religious rituals*. Certainly, such sequencing data can be obtained. However, how is one supposed to define, among the numerous infections, the strains of interest that not only cause an infection but also make individuals eager to participate in the ritual, if the rituals are not defined? In the article Panchin et al. have proposed an unsystematic range of suspicious rituals (circumcision in Judaism, kissing of holy relics in Christianity, Hindu side-roll, ablution and Hajj congregation in Mecca, and Ganges River bathing in Hinduism) without indication of an appropriate high-priority marker of religion behavior. In reality, such analysis must be performed more scrupulously.

In order to meet the falsifiability requirements, the biomeme hypothesis could be narrowed down and evaluated for a particular case (to test the problem for the particular conditions, as mathematicians say). As an example, one could evaluate a possible role of microbes in promoting Christian rituals. To meet Popper’s criterion, the target religious rituals of Christianity should possess features favorable for microbial transmission. We should consider only rituals with some other specific features such as high regularity, with a weekly or a monthly period; a large number and a high density of participants; no reasonable sanitary protection, and so on. Ideally, the given ritual(s) also would have analogues in other religions. From this point of view, two Christian rituals look to be the most appropriate: the public icon kissing, and the drinking from the Communion Chalice. Since both rituals are traditionally performed in churches, the frequency of church visits can be taken as a quantitative parameter of the religiosity. In this truncated form, the hypothesis is ready for testing.

By Panchin et al., one of the predictions from the biomeme hypothesis is the existence of a direct correlation between the religiosity in society and sanitation [6]: worsening of sanitary conditions should lead to increased number of people visiting church per month, and vice versa. However, history of Christianity shows a different regularity. As a prominent example, we can mention an atheistic outbreak in Russia (later USSR) in 1917–1939, when religion was severely restricted on ideological grounds after the October Revolution. It is noteworthy that during that time, due to Revolution and Civil War, conditions for microbe transmission were far more favorable than they are in the present day. Nevertheless, by 1939 only about 500 of the more than over 50,000 churches that functioned in 1917 remained open [12]. The persecution of religion resulted in plausible drops in the number of people visiting churches: once religion becomes a dangerous activity, the number of its active participants dramatically decreases without any bias to sanitary conditions in society. Thus, this example denies a correlation between sanitation rate and a number of individuals participating in Christian rituals. Similar situations have occurred throughout human history: repression against all kinds of Paganisms in early Europe, the persecutions of the Old Believers in medieval Russia, the consequences of the Cultural Revolution in China in the 1960s etc. In most of cases, a number of the active believers depend on authorities’ position toward a religion rather than sanitary conditions in societies.

The example above is just a particular interpretation of the hypothetical consequences of Panchin’s idea, which of course does not ultimately disprove the biomeme hypothesis in general. It illustrates however that the biomeme idea ought to be formulated more thoroughly and precisely to become testable, and hence to be admitted as a scientific hypothesis. Certainly, the metaphysical nature of the hypothesis does not deny its potential significance. The main message of this chapter is that the biomeme hypothesis cannot be assessed as a scientific hypothesis.

## Occam’s razor as another instrument to test the biomeme hypothesis

Regardless of its scientificity, the biomeme hypothesis can also be tested using Occam’s razor, a problem-solving approach introduced by the medieval philosopher William of Occam (Ockham). Occam’s Razor principle can be formulated as “The simplest solution to a complex problem is usually the correct one” (originally, “*Numquam ponenda est pluralitas sine necessitate*”, or “*Plurality should not be posited without necessity*”). Since Panchin’s idea provides an alternative explanation for the origin of religious rituals, it can be opposed by a conventional explanation of religion as a social phenomenon. Briefly, conventional theory of development of religious rituals can be described as follows:

- The very first peoples (most likely, in the Paleolithic and Neolithic eras) perceived all natural objects – forests, rivers, mountains, etc. – as live creatures with their specific behaviors, where natural disasters were attributed to nature’s angry mood. At this stage, rituals were devised to mitigate the dark moods of nature, analogous to how a weaker person might pacify a stronger one with gifts, stories, or entertainments. From these behaviors the first religious rituals appeared [13].
- Specialization in later civilizations led to a hierarchy and diversification in society. This process was also reflecting in religious institutes - the shamans, and later the priests, rabbis etc. appeared as interpreters and defenders of society from the threat of natural forces. Further divergence of society led to empowering of those positions. Religious representative were entrusted for the preservation and maintenance of accepted rituals, leading to their canonization [13,14].

Over time, many rituals lost their original meaning, and nowadays most people have stopped praying to the gods for rain. Nevertheless, the religious rituals gradually transformed into social institutions that bring people together to make them feel part of a group, which is a very important feeling for social organisms. The impact of religion and religious rituals in human culture so far remains unprecedented. A substantial body of sociological research suggests that religious beliefs are associated with better mental health as indicated by satisfaction with life, better mood, feeling of happiness, less depression, and less addiction among believers compared to non-believers [13-15]. Regarding this, Panchin et al. should be extremely cautious when assigning rational meaning to religious rituals as *useless activities without obvious benefits* [6].

Thus, we have two different explanations for the origin of religious rituals to compare: conventional sociologic one (“social”) and “microbial” hypothesis proposed by Panchin et al. Below I propose a few consequences to analyze, two of which describe religion behavior in the simplest way. For example, “social” theory logically interprets appearance of religious leaders; the “microbial” one can barely explain how microbes could induce a religious hierarchy as such.

Any religion entails hundreds of rituals, most of which do not facilitate transmission of microbes. Therefore, the majority of the rituals appear to be worthless from a microbial point of view. To explain this, the adepts of the “microbial” hypothesis have to separate religious rituals into two distinct groups: “promicrobial” rituals, which can facilitate infection transmission, and “neutral”, which bring no benefits to microbes. This immediately raises the question: do these two groups of rituals have different origins? The authors tried to explain this by introducing an idea of a symbiosis between informational memes (originated the “neutral” rituals) and biological organisms (driving “promicrobial” rituals) [6]. However, the term “symbiosis” means an interaction between two or more biological species, while the “meme” clearly is not. Theoretically, it is still possible to compose some microbiological explanation of coexistence of these rituals, but it seems to be a non-trivial problem. In turn, from a position of the “social” paradigm both “types” of rituals are interpreted without visible contradictions.

In terms of infection transmission there are no clear differences between religious and non-religious events. Besides organized religion, individuals form many other social connections and group according to their interests such as sport team fans, political movements, scientific or educational events, etc. Every non-religious group by default establishes some special rituals, and some of them have a great potential for microbial transmission. Indeed, numerous individuals crowd into public transports every morning, or attend night clubs every weekend, or theaters every month, etc. Even scientists have very dangerous rituals such as regular attendance at conferences, often with thousands of other people from different continents all concentrated for long periods of time in a small area. Thus, our life includes hundreds of non-religious behaviors (many of which one may qualify as rituals) and can potentially spread infections. From the “social” point of view, these rituals have the same origin as religious ones – people simply like to feel part of groups. However, if we consider a microbial origin of religious rituals, we should admit existence of microbial influence on non-religious rituals as well. Should we accept the idea that many of our social choices are managed by microbes, which manipulate our mind in order to support their reproduction? Apparently not, because the conventional interpretation of rituals sounds much simpler: our microbes nimbly use our habits to spread themselves under the favorable occasions without any conspiracy.

**Figure 1:**
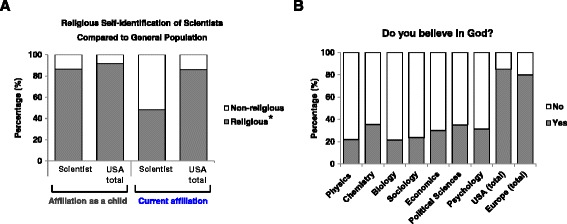
**Intelligence and religiosity: (A) –Religious self-identification of scientists compared to the general population at childhood, and adulthood; (B) – Ratio of the people answered “Yes” or “No” to the question “Do you believe in God?” among scientists, and non-scientists** **[** 17 **].** * - The parameter “Religious” includes several affiliations such as Protestant, Catholic, Jewish, Buddhist, Hindu, Muslim, Orthodox, etc. A detailed list of affiliations and percent values can be found in Table 4 in [17].

One more intriguing outcome from the hypothesis, which was mentioned by Panchin et al., was that there could be changes in the future religious practices of children who have been subjected to different antibiotic treatments during childhood [6]. Unfortunately, such data are absent in the literature. Nevertheless, some alternative data might be of interest to this point. Sociologists have evaluated the influence of religiosity of the families in which future scientists grew up (Figure 1A). Although at childhood the ratio between religious and non-religious families is similar in the both groups, in adulthood a much higher percentage of scientists than control group members become atheists regardless of the religious environment in early life. These findings seem hardly compatible with the infection-related origin, whereas social roots of religion recognize no contradiction in this.

Generally, the negative correlation between intelligence and religious beliefs is a popular topic in sociology, which can hardly be interpreted from the position of the “microbial” hypothesis. For example, meta-analysis of 63 studies has shown a significant negative association between intelligence and religiosity [16]. Figure 1B shows some numeric data adapted from a recent article [17]. Again, these data are poorly compatible with the prediction from the “microbial” hypothesis, because all scientists live among purported believers, while the microbes of religiosity are expected to be contagious by definition. Thus, scientists are expected to be infected by religion-promoting microbes roughly at the same rate as other people. Nevertheless, the percentage of believers among scientists is on average three-fold lower compared to the general population. These correlations are poorly compatible with the infection hypothesis, while psychology can explain them in a logical manner: individuals may eschew religious groups in accord with their own needs and interests.

The purpose of the aforesaid is to illustrate that the biomeme hypothesis looks to be far-fetched and redundant for explanation of some aspects of religious behavior. In other words, the “microbial” hypothesis fails to overpass the conventional “social” theory in terms of simplicity, and looks like an appropriate candidate to be cut off by Occam’s razor.

## Conclusion

Each of the approaches and arguments presented above is perhaps debatable. Nevertheless, together they force one to conclude that the microbial hypothesis can hardly explain the wide spread and perseverance of religious rituals whereas sociology, history, and psychology explain them in a simple and convincing manner.

Definitely, our microbiome can affect our mind and our rituals: suffice it to say, perhaps, that chronic infection triggers a daily ritual of swallowing medicine. However, we cannot blame microbes as specific causative agents of religious rituals. The microbes gingerly use these rituals for their transmission, but to the best of our understanding no conspiracy is involved.

## Reviewer’s comments

### Reviewer #1: Dr. Eugene Koonin, National Center for Biotechnology Information (NCBI, NLM, NIH, United States of America)

In this Comment, Starokadomskyy presents a critical analysis of the recent Biology Direct article by Panchin et al. that hypothesizes on the role of the microbiome in the origin of religious rituals. I agree with Starokadomskyy that, notwithstanding all the physiological importance of the microbiome and its likely behavioral effects, the microbial hypothesis of the origin of religious practices does not stand the test of the Occam’s razor. Simply put, at this stage, it is no more than wishful thinking.

This is not the place to seriously discuss the origin and evolution of religion but I nevertheless would like to make a brief remark on the first conclusion of the article, that the persistence of religion has a clear adaptive explanation at the social level. I believe that in this case, as generally in evolutionary biology, ultimate caution is required before adaptive scenarios are invoked. I suppose that the neutral explanation, namely religion as a selfish meme, is at least as plausible as the adaptive hypothesis. My own suspicion is that a trait that is as successful as religiosity cannot survive via the selfish or the adaptive route alone, but only through a combination of both.

Author’s response: I am grateful to Dr Koonin for his thoughtful review.

Since I am not a specialist in the questions of religion, I quoted the thought about adaptive functions of religion from several sociologic articles and books. However, the terms “adaptive function” and “adaptive role” indeed possess quite different meanings in Sociology and Evolutionary Biology. In order to prevent a misunderstanding, I rehashed my conclusions in a more accurate way, taking into account that the article is directed mostly to biologists.

### Reviewer #2: Neil R Smalheiser, University of Illinois at Chicago, US, First review

Within the scientific community there are many types of inter-scientist communications which (strictly speaking) do not fall within science per se yet which serve supporting roles. For example peer review is not a formal process specified in detail nor does it deals with objective observations nor is it reproducible which are all features of scientific investigation. Yet peer review is (or at least is intended to be) in the service of scientists and thus moves science forward. The formulation of speculative heuristic questions (What if “…”) is another example of a non-scientific process which can potentially enrich science by motivating scientists to think outside their comfort zone. What if? people don?t necessarily need to grow old and die? What if “aliens had contact with early civilizations”. What if “it were possible to go to the moon”. These are not and are not intended to be scientific hypotheses in the strict sense? they are generally not precisely formulated in terms of details or mechanisms or testable against pre-specified alternatives. Yet it cannot be denied that they may have value when they inspire scientists and stimulate thought.

Recently Biology Direct published an article by Panchin et al. which is marked as a Hypothesis article but which really is of the What if… variety. Given certain precedents that appear to have scientific support in the animal kingdom (e.g. Wolbachia bacteria are parasites that influence the behavior of their hosts in such a manner as to favor themselves) what if gut microbiota species can affect the behavior of humans too in such a way that it favors their spread to infect more humans? In particular they suggested that bacteria acted to facilitate the performance of religious rituals. This proposal is certainly amusing yet it does succeed in stimulating thought insofar it raises awareness that there may be many potential interactions between gut microbiota and humans that remain to be explored. Moreover it hits a ?hot button? by mentioning religion and thus was quite successful in attracting widespread attention as a meme itself!

A scientific hypothesis can be assessed in various ways for example in terms of the strength of the supporting evidence the plausibility of known alternatives and the impact of the finding if true. How should one assess a “What if Article’ A wise saying attributed to Warren McCulloch is: “Don’t bite my finger look where I am pointing.” The present paper has taken up the challenge (one is tempted to say fell for the bait) of providing critique and Comment on the Panchin et al. article. Unfortunately the author spends most of the time biting their fingers rather than looking where they are pointing.

Many of the criticisms which are discussed here are off-base because they deal with imprecision in the way that the authors formulated their proposal. For example the author points out that some religious rituals are infrequent (and so don’t spread germs very often per person) - yet that does not really matter since even if bathing in the Ganges is something done by an individual only once many individuals are bathing at any given time so it could (and does!) spread germs substantially throughout an ecosystem on an almost continual basis. Another example is that the author notes correctly that there may not be any fundamental distinction between religious and non-religious behaviors that spread microbes, yet that does not really matter either to the basic idea (do gut microbes influence human behavior to facilitate their own spread?).

Another off-base criticism is that the Panchin et al. proposal assumes that religious rituals must have no other meaning or significance OTHER THAN to spread microbes. It is true that Panchin et al. focused on the apparently meaningless rituals because those are in most need of an explanation but nothing in their proposal requires that ONLY meaningless rituals are affected. Certainly religion DOES have significance (across several dimensions) and the author feels that this therefore creates a problem for the microbial proposal. However microbes could certainly affect physiological systems that have their own functions. I don’t know much about Wolbachia (which served as one precedent) but I understand that it confers selective advantages on the infected host and alters sexual selection practices which certainly are not meaningless or without their own functional significance.

More importantly the author mis-understands what the Panchin et al. proposal is saying. They are NOT saying that microbes directly force humans to behave in certain ways like blind automatons but rather they introduce INFLUENCES that have the force of memes. Think of TV ads: They certainly influence human behavior sometimes powerfully sometimes ineffectively but always indirectly. Perhaps the strongest evidence raised by the author against the microbial proposal is that there is apparently an inverse relationship of “IQ/intelligence” and “religiosity”. Unfortunately neither “IQ/intelligence” or “religiosity” are well defined clearly measured or generally accepted hard-science concepts! Nor is it clear what the inverse relationship is supposed to mean. The author assumes that if everyone in the US is equally exposed to the microbes they should be equally susceptible to their effects [or else the proposal must be wrong]. However it is entirely possible that everyone is infected yet the less intelligent are more susceptible to being influenced by the microbes (even if everyone watched TV equally certain types of people are more susceptible to buying from infomercials).

The author also discusses the Panchin et al. proposal as if it were trying to explain religion or religious observance in general whereas they specifically denied this? In this context we would like to clarify that our hypothesis is not about religion in general but mostly about specific religious rituals that do not provide apparent benefits to those who perform them but facilitate microbial transmission.? The author does in fact note this disclaimer in the Background section yet ignores it in the following text.

The author points out that only half of scientists self-identify as religious which is less than the general population from which they arise. However again this is a statistic that can be interpreted in many different ways. The microbial proposal is not that religious affiliation is solely (or even partially) caused by microbes. The microbial influence may bias or alter the behaviors that a human already has rather than causing new behaviors de novo. Furthermore the author points out that the OVERT expression of religion can be suppressed in dictatorships. That is off-base for several reasons. First many things that are suppressed in dictatorships continue under the surface (including expression of religion? think of the Jewish Marranos for one). Secondly as mentioned above this criticism assumes that microbes are supposed to be forcing behavior rather than having subtle effects on behaviors which I think is the heart of the Panchin et al. proposal.

Finally the author greatly over-states what science knows about religion in general. I strongly disagree that sociology biology or any other science can “explain” religious belief neither in a “simple and convincing manner” (p. 8) nor in any other manner.

Could there be valid and strong criticisms of the Panchin et al. proposal? Yes I believe so! I am not trying to sound like its champion. However the present version of this manuscript primarily attacks its vagueness? whereas vagueness is perhaps its greatest strength! Instead one could ask critically whether the biological analogies which motivated the proposal (e.g. Wolbachia and others) might be false or misleading analogies. Or one could more properly identify strong predictions of the proposal which are contrary to actual observations. One could argue that known memes do not have the specificity or power that would be needed to exert the type of effects attributed to microbes in the proposal. One could argue that gut flora turn over too rapidly are too variable and unstable within individuals over time or have other properties that make behavioral control unlikely. One could point out that “irrational ritualistic behavioral activities” are equally or more characteristic of OCD (obsessive-compulsive disorder) than religious observance so that microbes should therefore be expected to affect the former behaviors as well as the latter? yet possibly the features of OCD may be at odds with those needed to fit their model.

Midichlorians may or may not have provided the original inspiration but certainly the Panchin et al. proposal had a light touch and a playful literate and panoramic perspective that stands astride science and science fiction. Any Comment on their paper should acknowledge this and take this into account.

Quality of written English: Acceptable.

Author’s response: I am grateful to Dr Smalheiser for the detailed review. In order to make my points more obvious, I restructured my comment on the “biomeme hypothesis”. To me, vagueness in scientific articles is not acceptable, because it turns a scientific hypothesis into an irrefutable fiction. Therefore, I am “biting the finger” because it is pointing nowhere.

However, the position of the reviewer is also clear to me, and certainly I accept it although it is quite opposite to mine own. This conflict can be illustrated by one sentence from the article “How many scientists does it take to change a paradigm?”:

“…*one cannot simply weigh all of the evidence because each side rejects the type of evidence that the other side accepts, and regards the alternative explanation not merely as wrong but as ridiculous or nonsensical*…” [18].

Nevertheless, reviewer’s detailed comment allowed me to understand several flaws in the structure of my own manuscript. I tried to rewrite it in order to present my position in more clear and scientific way.

Regarding the acknowledgment of Panchin’s idea, I believe that my responding manuscript is already indicating my acknowledgement of his idea, otherwise why would I spend so much time for it? In view of this, I believe that my comments highlight the flaws in the Panchin et al. theoretical speculations, which they may somehow use to enforce this hypothesis in the future (although I am not sure if it is possible).

### Reviewer #2: Neil R Smalheiser, University of Illinois at Chicago, US, Second review

I don’t think that further revision is warranted. I do have a cautionary side-note about Occam’s Razor, though. I was just teaching a lecture on microRNAs to graduate students, and one student rightly pointed out that microRNAs fail the Occam’s Razor test. That is, at the time that the first microRNA was discovered, there were already multiple systems known to regulate protein translation and mRNA stability, and no one had predicted the need for yet another system. Even now, we can say that microRNAs play roles in this-and-that, but I am not sure that we can assert that it would be impossible to model a cell without including microRNAs. Having redundant regulatory systems seems to be the norm in biology, which confounds Occam, at least to some extent.

Quality of written English: Needs some language corrections before being published.

Author’s response: I am thankful to Dr Smalheiser for his comment. Indeed, Occam’s razor is an old-school instrument, and it has been applied simply because more contemporary scientific approaches are barely applicable in this case. Regarding your example, RNAi phenomenon meets Popper’s criterion, and thus can be tested by current scientific techniques. Hence, proof of RNAi does not necessitate the appliance of medieval scientific approaches.

### Reviewer #3: Etienne Joly, CNRS, France, First review

This manuscript is not a scientific paper but a comment on a highly speculative hypothesis published earlier this year in the same journal by Panchin et al. proposing that some religious rituals could be induced by the influence of microorganisms on the human brain for the sole sake of favoring the spread of those microbes.

The three referees of the Panchin paper had all underlined that this hypothesis despite being presented in a pleasant and thought-provoking manner lacked any scientific grounds.

Regarding this current manuscript I must first say that I am in complete agreement with all the comments made by Eugene Koonin. In short I feel that the main value of this manuscript lies in it’s title: Following the principle of Occam’s razor there indeed seems to be no need to call upon microbial influences to explain religious behaviors.

Sadly this point about parsimony is not developed any further in the manuscript and in my eyes the paper actually falls in the same vein as the paper it criticizes: it makes for a reasonably entertaining read but does not contain any solid scientific argument. For example I simply cannot make sense of two arguments listed as 3 and 4 in the conclusion. Why should the fact that there is a negative correlation between religiosity and either higher IQs or authoritative banning of religion be arguments against the idea that some microbes could promote human religious behaviors that favor the spreading of those microbes?

All in all I am simply left wondering whether those two papers really belong in a scientific journal.

For the author’s benefit I also spotted a score of typing mistakes and grammatical loose ends which can be found in the attached pdf.

Quality of written English: Needs some language corrections before being published.

Author’s response: I am grateful to Dr Joly for his concise review. In order to address his critique, I added detailed descriptions of criteria for a scientific hypothesis (Popper’s principle), and rewrite with additional details the chapter devoted Occam’s razor criterion. Initially, I tried to avoid formal scientific language; however the reviewer’s commentary indicated that this was not the right approach. I hope, that this time my manuscript would be accepted as more scientific rather than entertaining one.

### Reviewer #3: Etienne Joly, CNRS, France, Second review

This manuscript is not a scientific paper, but a comment on a highly speculative hypothesis published in the same journal by Panchin et al. proposing that some religious rituals could be induced by the influence of microorganisms on the human brain for the sole sake of favouring the spread of those microbes.

The three referees of the Panchin paper had all underlined that this hypothesis, despite being presented in a pleasant and thought-provoking manner, lacked any scientific grounds.

Sadly, this manuscript actually falls in the same vein as the paper it criticizes in that it dwells at length on woolly considerations and does not contain any solid scientific argument. All in all, I am simply left wondering whether those two papers really belong in a scientific journal?

Quality of written English: Acceptable.

Author’s response: I thank Dr Joly for his careful review, but I must stress my view that it is almost impossible to prove or disprove, with scientific arguments, a hypothesis that is non-scientific. Actually, this point of view is one I am pursuing in my manuscript (please, see Popper chapter).
